# Feasibility and preliminary effects of an individually customizable ecological momentary stress management intervention: A mixed methods pilot study

**DOI:** 10.1016/j.invent.2025.100901

**Published:** 2026-01-13

**Authors:** Hannah Tschenett, Aljoscha Dreisörner, Katrin Schäfer, Ricarda Mewes, Urs M. Nater

**Affiliations:** a Department of Clinical and Health Psychology, Faculty of Psychology, University of Vienna, Liebiggasse 5, 1010, Vienna, Austria; b Research Platform “The Stress of Life (SOLE) — Processes and Mechanisms Underlying Everyday Life Stress”, University of Vienna, Liebiggasse 5, 1010, Vienna, Austria; c Institute of Psychology, University of Klagenfurt, Universitätsstraße 65-67, 9020, Klagenfurt am Wörthersee, Austria; d Department of Social and Preventive Medicine, Center for Public Health, Medical University of Vienna, Kinderspitalgasse 15, 1090, Vienna, Austria

**Keywords:** Stress management, Ecological momentary intervention, Digital intervention, Feasibility, Stress, Fatigue

## Abstract

While many interventions are known to reduce stress, evidence-based stress management interventions that can be applied during stressful events in everyday life are lacking. This pilot study investigated an ecological momentary intervention for everyday stress management, comprising eight modules (e.g., relaxation, music listening), in terms of its feasibility, its immediate effects following stressful events, and its pre- to post-intervention effects. Over 45 days, undergraduate psychology students (*N* = 27, 21.6 ± 1.9 years, 74 % female) completed momentary assessments four times daily and whenever they experienced stress. During the 35-day intervention period (days 6–40), participants were intra-individually randomized to either use the intervention (5–20 min intervention use) or not use the intervention (continuing with usual activities) after indicating a stressful event. Additionally, they used the intervention as needed in their daily lives. The feasibility of the intervention was indicated by no drop-outs, high usage rates and positive reports in the acceptability questionnaire and semi-structured group interviews, while lower compliance with momentary assessments indicates reduced feasibility of the study design. After using the intervention in response to stressful events, participants reported significantly decreased stress and negative affect. Moreover, participants showed improvements in stress, mindfulness, and self-compassion post-intervention. Our pilot findings suggest that the intervention is feasible and indicate reductions in event-related and daily stress in everyday life.

## Highlights

• Ecological momentary intervention for everyday stress management is feasible.
• Using the intervention during stressful events reduced stress and negative affect.
• Participants reported lower daily stress levels after the five-week intervention.

## 1 Introduction

Stress is a well-known experience of everyday life. Encountering and dealing with stressors is a fundamental and adaptive part of human existence. While stressful major life events (e.g., the loss of a loved one) generally occur only occasionally in a person's life, smaller stressful events (e.g., being stuck in traffic, social conflicts, time pressure)—so-called daily hassles—can arise on an (almost) daily basis. The human organism is capable of adjusting to and adaptively regulating biological stress when confronted with threats and challenges (Cannon, 1932). However, in the case of overexposure to stressors or experiencing stressors over a prolonged period of time, this adaptive regulatory process becomes more challenging or even impossible, in turn potentially leading to a dysregulation of bodily stress systems and (sub-)chronic stress (Chrousos, 2009). High levels of ongoing stress have been shown to be a risk factor for poor health (O'Connor et al., 2021) and have been related to different somatic and mental health symptoms (e.g., fatigue, Doerr et al., 2021) and conditions (Schneiderman et al., 2005).

Therefore, interventions are needed that aim to reduce the negative consequences of ongoing or excessive stress. Research has already demonstrated the effectiveness of various interventions in terms of reducing ongoing or momentary stress and enhancing somatic and mental health. For instance, besides interventions derived from cognitive behavioral therapy (CBT), such as time management (Aeon et al., 2021) or problem-solving training (Ebert et al., 2014), mindfulness-based meditation and relaxation have been found to improve stress and various health outcomes in different populations and settings (Goldberg et al., 2021; Pickett et al., 2022; Sedlmeier et al., 2012). Moreover, yoga interventions (Breedvelt et al., 2019; Pascoe et al., 2017; Wang and Szabo, 2020), music listening (De Witte et al., 2020), self-compassion interventions (Ferrari et al., 2019), journaling about stressful events (Ullrich and Lutgendorf, 2002), and receiving social support (Ditzen and Heinrichs, 2014) have all been shown to reduce stress.

Additionally, combining different stress reduction techniques within one program has proven to be beneficial (e.g., mindfulness-based stress reduction (MBSR) program (Kabat-Zinn, 2013)). Many of the programs that combine techniques require participants to learn all of the included techniques and train the newly learned skills in a structured way. On the other hand, there are also non-structured interventions, in which participants are able to choose the strategies and activities they find suitable, interesting, and potentially beneficial (Huberty et al., 2019), hereinafter referred to as “individually customizable interventions”. These interventions take participants' individual needs and preferences into account, and may consequently enhance an intervention's acceptability, adherence, and potentially also outcomes (Perski and Short, 2021).

Individually customizable interventions can be effectively implemented using a digital format, which offers various advantages such as easy and low-threshold access, low cost, and easy integration into everyday life (Mrazek et al., 2019). The feasibility and effectiveness of many digitally delivered stress reduction activities have already been demonstrated, including yoga (Brosnan et al., 2021; Chang et al., 2022) or mindfulness-based interventions (Jayawardene et al., 2017; Schulte-Frankenfeld and Trautwein, 2022; Xu et al., 2022).

Ecological momentary interventions (EMIs) are a special case of digitally delivered interventions provided in everyday life, i.e., in real time and in natural settings (Heron and Smyth, 2010). Beyond these characteristics, EMIs also allow for the assessment of dynamic as well as immediate effects without recall bias (Trull and Ebner-Priemer, 2013). Furthermore, EMIs enable the delivery of customized coping strategies during particularly stressful events in everyday life, when they are needed the most. While studies have shown that EMIs can effectively reduce stress (Schulte-Frankenfeld and Trautwein, 2022; Versluis et al., 2016), preliminary research further indicates that EMIs are feasible *during* stressful events (Feneberg and Nater, 2022) and reduce both stress levels and the frequency of stressful events (Smyth and Heron, 2016). However, despite these promising initial findings, research on the implementation and effectiveness of EMIs that offer stress reduction activities during stressful events is still scarce.

### 1.1 The present study

To advance the literature on stress management interventions for everyday life, we developed an EMI called EPIGRAM (**e**Health **p**revent**i**on pro**gram**), which is an individually customizable stress management intervention that includes a variety of evidence-based stress reduction components and strategies presented as individually selectable activities embedded in eight modules and a daily diary. Specifically, EPIGRAM aims to 1) train stress management skills in the longer term as a self-administered preventive health intervention, and 2) provide tools for regulating stress during acute stressful events in everyday life.

The present mixed methods pilot study examined the feasibility and potential benefits of EPIGRAM in a sample of undergraduate psychology students using an intra-individual randomized design. University students have reported elevated stress levels, both prior to and during the COVID-19 pandemic (Elmer et al., 2020; Ochnik et al., 2021; Voltmer et al., 2021). High levels of stress in students have been shown to predict negative health outcomes (i.e., symptoms of depression, Margraf et al., 2020). Therefore, training stress management skills is particularly important for university students and may benefit their health in the long term.

Our primary goal was to investigate the feasibility of the intervention EPIGRAM in everyday life and the study design (primary outcome). Additionally, we hypothesized that EPIGRAM would be effective in reducing momentary subjective stress following acute stressful events (immediate effects, H_1_). We further hypothesized that EPIGRAM would be effective as a stress management program, reducing average stress levels in everyday life from baseline to post-intervention (intermediate effects, H_2_). As fatigue has been related to momentary stress and is prominent in various stress-related somatic and mental health disorders (e.g., depressive disorders, somatic symptom disorder (Doerr et al., 2021)), we investigated H_1_ and H_2_ analogously for fatigue (immediate effects, H_3_; intermediate effects, H_4_). From an exploratory perspective, we analyzed the influence of EPIGRAM on positive and negative affect following acute stressful events as indicators of positive and negative mental health. Finally, given that most of the programs and literature informing EPIGRAM assume mindfulness and self-compassion as underlying principles (Neff and Germer, 2018; O'Shea et al., 2022; Segal et al., 2015; Kaluza, 2023), we conducted an exploratory analysis of EPIGRAM's effects on mindfulness and self-compassion from baseline to the post-intervention period.

## 2 Methods

### 2.1 Participants

Based on sample sizes in similar EMI studies (Marciniak et al., 2020), we aimed to include at least 20 participants (with 180 scheduled data entries per participant) to allow for initial analyses regarding the potential impact of EPIGRAM in reducing immediate and intermediate stress (Snijders, 2005). In total, 27 undergraduate psychology students were recruited from a course on research methods at the University of Vienna. Participants were provided with a brief face-to-face introduction to the study during one of the course sessions, after which they were given the option to participate in the study. There were no inclusion or exclusion criteria, and neither the study participation itself nor compliance with the study affected students' grades in any way. All participants participated voluntarily in the study and were informed orally and in writing about the study and provided written informed consent. Participants could terminate their participation at any time without negative consequences.

The overarching project, the “Vienna Health in Everyday Life” (V-HEAL) study, was approved by the Ethics Committee of the University of Vienna (reference number 00821, 17th June 2022) and the present study was preregistered on the Open Science Framework (doi:10.17605/OSF.IO/23Z9P). The study was conducted in accordance with the ethical standards of the institutional ethics committee and the 1964 Helsinki declaration.

### 2.2 Procedure

**Fig. 1:**
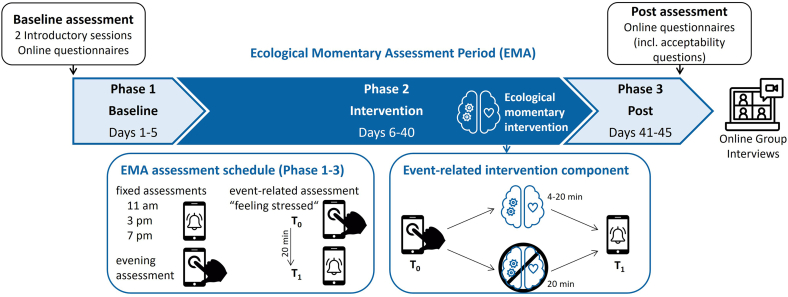
*Study design.* The alarm icon indicates automatically initiated assessments, the hand icon indicates self-initiated assessments. The brain icon indicates the availability of the ecological momentary intervention (EPIGRAM modules). For the event-related intervention component, the upper path indicates the intervention condition, the lower path indicates the control condition. Icons provided by https://www.flaticon.com/.

An overview of the study design is shown in Fig. 1. Data collection took place from October 24th, 2022, to January 30th, 2023. At baseline, participants completed a battery of online questionnaires (see below under ‘Measures’). The ecological momentary assessment (EMA) lasted for 45 days and was divided into three phases: a 5-day baseline phase (Phase 1), a 35-day intervention phase (Phase 2), and a 5-day post phase (Phase 3). During all phases, participants answered questions via their study app at three fixed assessment time points per day (11 am, 3 pm, 7 pm) and completed a self-initiated evening assessment before going to bed. Daily measures included momentary stress and fatigue, as well as mindfulness and self-compassion in the evening assessment. Participants were also instructed to self-initiate event-related data entries whenever they felt stressed (defined as “experiencing a situation that is personally important and unpleasant and that seems hardly or not at all manageable through one's own efforts at the moment,” based on the transactional stress theory), which were followed by event-related questions immediately (T_0_) and 20 min after the entry (T_1_). Event-related measures included momentary stress, fatigue, and positive and negative affect. In Phase 2, when participants reported a stressful event (T_0_), those events were intra-individually randomized using block randomization (block length: 2; allocation ratio: 1:1) to one of two conditions: (1) use EPIGRAM as an event-related intervention after reporting the stressful event (intervention use), or (2) continue usual activities (no intervention use). For condition (1), participants were prompted once to select a suitable intervention activity, with no active option to dismiss or snooze the intervention. However, participants could choose not to engage by not selecting or skipping the activity. Participants were informed in advance about all available options. Participants answered the event-related questions immediately (T_0_) and either 20 min after the initial entry (no intervention use) or 20 min after the intervention was completed (intervention use) (T_1_). Additionally, during Phase 2, participants had access to the EPIGRAM modules in order to train their stress management skills and were able to complete a daily diary. Throughout the EMA period, participants had the opportunity to discuss any questions and problems in weekly question and answer group sessions, moderated by two study team members (psychology Master's students). Moreover, they were able to send individual inquiries via email or the messaging tool of the study app to receive personalized on-demand support from the study team. Participants also received nine reminders through the messaging tool, aimed at providing support and motivation.

After the 5-day post phase (Phase 3), participants completed a second battery of online questionnaires, including a questionnaire assessing the acceptability of the intervention (see below under ‘Measures’), and participated in an online group interview to discuss the feasibility and usability of the intervention.

### 2.3 Intervention

In one introductory session, participants received an introduction to stress and stress management skills, information about the different modules and activities, and instructions on how to use the app. In addition, the introductory sessions served to address open questions regarding the intervention and study procedure. The session lasted 90 min and was conducted face-to-face with all study participants. Relevant information was presented on slides, which were made available to the participants via their e-learning platform Moodle (Moodle Pty Ltd). Participants were further provided with a study manual summarizing this information. The intervention was accessed via the study app (MovisensXS, Movisens GmbH) installed on participants' study smartphones (four out of the 27 participants used their private smartphones). The EPIGRAM modules available for the self-initiated intervention usage are presented in Table 1. The research team did not recommend a sequence of modules or frequency of usage. However, participants were encouraged to engage with the app as frequently as possible and to explore different modules. When participants used EPIGRAM as an event-related intervention after reporting stressful events (intervention use), they had the option to choose between a short (5–10 min) and a long activity (10–20 min). Depending on the chosen duration, participants were presented with a list of possible activities suitable for regulating acute stress reactions in everyday life (for details see Table 1).

**Table 1:** Description of EPIGRAM modules.

Module	Content					Event-related intervention	
	Name	Format	Duration (min)	Description	Reference	Short activity	Long activity
1) Short-term stress management strategies	Psychoeducational introduction	Text		Strategy based on the 4A strategy by Kaluza (2018), including the steps 1) acceptance, 2) cool down (implemented via the two audio-guided activities), 3) analysis, and 4) action vs. distraction	(Kaluza, 2018, p. 191–193)		
	Breathing meditation	Audio^1^, text	6	Adapted breathing space meditation from mindfulness-based cognitive therapy; focus on the breath; including breathing technique (inhaling through the nose for two seconds, exhaling through the mouth for six seconds)	(Segal et al., 2015, p. 224)	x	
	Progressive muscle relaxation	Audio, text	8	Short version of progressive muscle relaxation; four muscle groups: 1) arms, 2) head, 3) torso, and 4) legs	(Kaluza, 2018, p. 107)	x	
2) Meditation and relaxation	Psychoeducational introduction	Text		Selection of different meditation and relaxation activities to enhance relaxation			
	Body scan	Audio	16	Adapted body scan version from mindfulness-based cognitive therapy; focus on different body parts and arising bodily sensations as well as breath	(Segal et al., 2015, p. 146)		x
	Breathing meditation	Audio	11	Adapted breathing meditation from mindfulness-based cognitive therapy; focus on natural breathing	(Segal et al., 2015, p. 199)		x
	Focus meditation (sounds & thoughts)	Audio	13	Adapted sitting meditation from mindfulness-based cognitive therapy; focus on sounds, thoughts, and breath	(Segal et al., 2015, p. 250)		x
	Loving kindness meditation	Audio	12	Adapted sitting meditation from Mindful Self-Compassion; self-soothing touch, breathing meditation, mental recital of affirmations towards 1) a loved one, 2) a loved one and oneself, 3) oneself	(Neff & Germer, 2018, p. 65)		x
	Progressive muscle relaxation	Audio	18	Long version of progressive muscle relaxation; four muscle groups: 1) arms and hands, 2) feet, legs, buttocks, 3) head and face, and 4) shoulders, back, chest, stomach	(Kaluza, 2018, p. 98–101)		x
3) Music and nature sounds	Psychoeducational introduction	Text		Selection of self-selected positive/relaxing music and nature sounds to enhance relaxation			
	Personal study playlist	Audio^2^	20	Study playlist with 20–30 self-selected music pieces (with or without vocals) that are experienced as positive, relaxing, and/or calming^4^	(Feneberg and Nater, 2022)		x
	Nature sound – Gentle forest rain	Audio	20	Nature soundtrack of a gentle forest rain	Sound effects from https://pixabay.com/de/sound-effects/, looped with GarageBand (mobile app, iTunes App Store)		x
	Nature sound – Thunderstorm at dusk	Audio	20	Nature soundtrack of a thunderstorm at dusk			x
	Nature sound – Summer day in the forest with stream	Audio	20	Nature soundtrack of a summer day in the forest with stream			x
	Nature sound – Night ambience with crickets chirping	Audio	20	Nature soundtrack of a night ambience with crickets chirping			x
	Nature sound — beach day with seagulls	Audio	20	Nature soundtrack of a beach day with seagulls			x
4) Yoga	Psychoeducational introduction	Text		Strategy based on operational definition of yoga by O'Shea et al. (2022) including the core components: a) physical postures (asana), b) breath regulation/activity (pranayama), c) relaxation/meditation (dhyana). Sessions are structured in three phases of modern postural yoga as described by De Michelis (2005): 1) introduction/arrival, 2) practice, 3) final relaxation. Type of yoga (vinyasa) is reported to be most commonly practiced online (Brinsley et al., 2021).	(O'Shea et al., 2022; De Michelis, 2005; Brinsley et al., 2021)		
	Yoga session 1	Video^3^	24	Focus on basic movements Breathing activity: Bhramari (Bumblebee Breath)	(O'Shea et al., 2022; De Michelis, 2005; Brinsley et al., 2021)		
	Yoga session 2	Video^3^	25	Focus on core activation Breathing activity: Sama Vritti (Equal Breath)			
	Yoga session 3	Video^3^	25	Focus on stability and standing postures Breathing activity: Ujjayi (Victorious Breath)			
	Yoga session 4	Video^3^	29	Focus on upper body and arms Breathing activity: Nadi Shodana (Alternate Nostril Breathing)			
	Yoga session 5	Video^3^	24	Focus on hips Breathing activity: Ujjayi (Victorius Breath) and Sama Vritti (Equal Breath)			
5) Problem solving	Psychoeducational introduction	Text		Strategy based on the module problem solving by Kaluza (2018), presenting two activities helping to systematically analyze a problem/stressful situation and to formulate and implement new solution approaches	(Kaluza, 2018, p. 140–157)		
	Problem-solving training	Text, writing	∼30	Problem solving training in five steps: 1) analysis of the stressor, 2) brainstorming of possible solution approaches, 3) selection of suitable solution approaches, 4) development of an action plan, 5) integration and evaluation	(Kaluza, 2018, p. 140–157)		
	Imagery: successful problem mastery	Audio	4	Imagery activity based on the module mental training by Kaluza (2018) as mental preparation for the successful mastery of problem/stressful situations	(Kaluza, 2012, p. 134)	x	
6) Time management	Psychoeducational introduction	Text		Strategy based on the supplementary module time management by Kaluza (2018), featuring three different activities training goal setting, structuring time and prioritizing tasks as well as planning positive activities	(Kaluza, 2018, p. 190–191)		
	Future vision	Audio, text, writing	∼15	Imagery activity based on the module problem solving by Kaluza (2018); imagining the time after having solved the next planned step in the future, supporting the development of positive future visions; accompanied by a writing activity leading to the formulation and planning of specific goals	(Kaluza, 2012, p. 113)		
	Prioritizing	Text, writing	∼15	Psychoeducational text based on the module problem solving by Kaluza (2018); introducing the Eisenhower matrix; accompanied by a writing task helping to prioritize upcoming tasks according to the matrix	(Kaluza, 2012, p. 115–117)		
	Planning positive activities	Text, writing	∼10	Writing activity based on the module enjoyment in everyday life by Kaluza (2018) leading to a specific plan to implement neglected positive activities	(Kaluza, 2018, p. 169–170)		
7) Social support	Psychoeducational introduction	Text		Strategy based on the supplementary module social support by Kaluza (2018) with activities promoting reflection on the current social network and ideas to maintain and expand it to enhance social support	(Kaluza, 2018, p. 183–185)		
	My social network	Text, writing	∼20	Drawing/writing activity based on the supplementary module social support by Kaluza (2018) to depict the current social network and identify social relationships one would like to deepen	(Kaluza, 2018, p. 183–184)		
	Practical tips for maintaining social contacts	Text	∼5	List of ideas on how to maintain and expand social networks	(Kaluza, 2018, p. 185)		
8) Self-compassion	Psychoeducational introduction	Text		Selection of different activities and guided meditations from Neff and Germer (2018), introducing self-compassion, offering activities on developing self-compassion, and providing strategies for coping with stressful events	(Neff and Germer, 2018)		
	How do I treat a valued person?	Text, writing	∼15	Writing activity based on Neff and Germer (2018); recall of a time when a friend was struggling, how participants would react towards the friend, then flipping the perspective focusing on how they treat themselves, focus on the differences between treatment of others and oneself	Translated from MSC program (Neff & Germer, 2018, p. 12)		
	Hand on heart	Audio	7	Guided sitting or lying-down meditation focusing self-soothing touch, from Neff and Germer (2018) and Dreisoerner et al. (2021a); self-calming gestures such as hands on heart or abdomen, self-embracing, or holding of the face, focus on warmth, breathing, and pressure	Dreisoerner et al. (2021b), translated from MSC program (Neff & Germer, 2018, p. 33)	x	
	Letter of self-compassion	Text, writing	∼20	Writing activity from Neff and Germer (2018); writing a letter from the point of view of a wise, loving, and compassionate friend, recalling perceived inadequacy and offering compassion, acceptance, and kindness	Translated from MSC program (Neff & Germer, 2018, p. 84)		
	Appreciation to the self	Audio	7	Guided sitting or lying-down meditation based on Neff and Germer (2018); focusing on three to five good qualities, visualizing them, how they have helped others, feeling gratitude for these qualities	Translated from MSC program by (Neff & Germer, 2018, p. 167)	x	
Extra: daily diary	Psychoeducational introduction	Text		Daily reflection on positive and negative/stressful experiences to support active awareness and the detection of behavioral patterns as a first step to change behavior, based on the daily review of positive experiences as part of the module enjoyment in everyday life by Kaluza (2012) and the stress-awareness diary by Davis et al. (2019)	(De Michelis, 2005, p. 21–24; Kaluza, 2012, p. 163)		
	Review stressful and positive experiences of the day	Text, writing^5^		Writing activity to reflect on positive and negative/stressful experiences of the day; description of positive/negative situations and associated thoughts/feelings/behaviors; positive visualization of the upcoming day	(De Michelis, 2005, p. 119; Kaluza, 2012, p. 163)		

1 All audio-guided activities, meditations, and yoga videos were recorded by an external voiceover artist on a professional cardioid condenser microphone (Maono, AM-PM421) and edited using GarageBand (mobile app, iTunes App Store) and Audacity (free software, https://www.audacityteam.org/).

2 Personal study playlists were not played through the app but via different music-listening apps used by the participants (e.g., Spotify).

3 Yoga videos were not played through the app but stored as videos on participants' (study) smartphones and played via linking the video with the app. Scripts were written by certified yoga teachers with 200 h of training (Yoga Alliance Registered Yoga Teacher RYT® 200).

4 Participants were instructed to listen to the study playlist only through the app during the whole study period, to prevent confounding effects.

5 Participants could fill out the diary in paper-and-pencil format in the manual, within the app, or in another form preferred by the participants. The module ‘short-term stress management strategies’ is available as an exemplary module on OSF, Suppl. 2.

### 2.4 Measures

#### 2.4.1 Baseline questionnaires

Online questionnaires were completed via Unipark (Questback GmbH; see OSF Suppl. 4 for all administered questionnaires) and were used to describe the sample in terms of their sociodemographic characteristics, general mental health, as well as ongoing stress and fatigue.

In addition to sociodemographic questions, we used the German version of the Patient Health Questionnaire (PHQ-D, Löwe et al., 2002) to assess participants' mental health. Two sum scores were calculated, with higher scores indicating a higher symptom burden: the PHQ-9 for depression (range: 0–27, *α* = 0.80) and the PHQ-15 for somatic symptoms (range: 0–30, *α* = 0.80).

Ongoing stress within the last month was measured using the German 10-item version of the Perceived Stress Scale (PSS-10, Klein et al., 2016). Items were rated on a 5-point Likert scale from 0 (*never*) to 4 (*very often*). The sum score ranges from 0 to 40, with higher scores indicating higher levels of chronic stress (*α* = 0.87).

Ongoing fatigue was assessed using the German version of the Multidimensional Fatigue Inventory (MFI, Schwarz et al., 2003). The inventory consists of 20 items rated on a 5-point Likert scale ranging from 1 (*yes, that is true*) to 5 (*no, that is not true*). Sum scores for the five subscales of the MFI were calculated (range: 4–20): general fatigue (*α* = 0.87), physical fatigue (*α* = 0.87), reduced activity (*α* = 0.78), reduced motivation (*α* = 0.75), mental fatigue (*α* = 0.85). Higher sum scores indicate higher levels of fatigue.

#### 2.4.2 Feasibility

##### 2.4.2.1 Usage and compliance

Usage rates were defined as the number/percentage of activities (within modules) logged by the participants during the intervention phase. Compliance with the assessment schedule was defined as the number/percentage of logged fixed assessments (11 am, 3 pm, 7 pm, self-initiated evening assessment) in all study phases. All data were retrieved from the study app, with the exception of the dropout rate.

##### 2.4.2.2 Usability and participant satisfaction

As a first indicator of acceptability, we assessed the dropout rate, defined as the number/percentage of participants who terminated their study participation prematurely. The usability of and satisfaction with the intervention were assessed using the acceptability questionnaire and group interviews (six to eight participants each). Acceptability (as defined by Sekhon et al., 2017) of the intervention was assessed in the online post questionnaire. The included items derive from existing scales adapted for the purpose of the present study (Brooke, 1996; Loo Gee et al., 2018; Lund, 2001) and self-developed items by the study team (see OSF Suppl. 4). Participants rated the items on a 5-point Likert scale ranging from 1 (*strongly disagree*) to 5 (*strongly agree*), or on a dichotomous scale (yes/no).

For the semi-structured online group interviews (via Zoom video calls, Zoom Video Communications Inc.), participants were randomly assigned to one of four interviews*.* Two study team members (psychology Master's students) led the interviews; one as observer, one as an active host. The utilized interview guide (see OSF Suppl. 3) covered three main questions: The first question explored participants' experiences with EPIGRAM in terms of its goal to a) improve long-term stress management skills and b) offer coping techniques for acute stressful events. The second question assessed the perceived helpfulness, structure, and design of two modules, which differed for each group in order to obtain specific information about each module. The third question focused on participants' ratings of the applicability of the app and the integration of EPIGRAM in their everyday lives. Each main question was supplemented with optional in-depth follow-up questions. The interviews were audio-recorded via Zoom and lasted between 61 and 83 min.

Feasibility of the study design was defined based on compliance with the assessment schedule, selected acceptability questionnaire items (e.g., data entries, alarms, and item-specific questions), and parts of the group interview (2 of 13 subcategories). Feasibility of the intervention was assessed using usage rates, dropout rates, the remaining acceptability questionnaire items, and the group interviews (9 of 13 subcategories).

#### 2.4.3 Momentary and daily measures

EMA measures were completed using the study app (see OSF Suppl. 5 for all administered EMA items). Participants reported their momentary subjective levels of stress (“At the moment I feel stressed”) and fatigue (“At the moment I feel fatigued”) on a unipolar visual analogue scale ranging from 0 (*not at all*) to 100 (*very much*). These items were completed at each EMA assessment time point (fixed and event-related assessments).

Positive and negative affect were assessed within the event-related assessments (T_0_ and T_1_) using six items from the German version of the Positive and Negative Affect Schedule (PANAS, Krohne et al., 1996). Participants rated the items on a 5-point Likert scale ranging from 1 (*not at all*) to 5 (*very much*). The items for positive affect were “active”, “enthusiastic”, and “determined”, while the items for negative affect were “scared”, “nervous”, and “afraid”.

Daily mindfulness and self-compassion were assessed within the daily evening assessment. Mindfulness was measured using two items adapted from the revised version of the Cognitive and Affective Mindfulness Scale (CAMS-R, Feldman et al., 2007). The original response format was adapted to a 4-point Likert scale ranging from 1 (*strongly disagree*) to 4 (*strongly agree*). Daily mean scores of the two items were calculated (*α* = 0.66). Self-compassion was assessed using six items from the long version of the State Self-Compassion Scale (SSCS-L, Neff et al., 2021). We selected one item from each of the six factors, choosing those with the highest factor loading within their respective factors. Participants rated the six items on a 5-point Likert scale ranging from 1 (*almost never*) to 5 (*almost always*). Daily mean scores of all items were calculated (*α* = 0.72).

The three fixed assessment time points included 21 items at 11 am (average response time: 43 s ± 28 s, range: 15 s–4 min 48 s), 15 items at 3 pm and 7 pm (45 s ± 34 s, 15 s–6 min 17 s), and 49 items at the event-related evening assessment conducted before bedtime (2 min 45 s ± 1 min 30 s, 43 s–12 min 8 s). The event-related assessments following stressful events included 24 items at T_0_ (2 min 11 s ± 59 s, 45 s–7 min 12 s), 22 and 21 items at T_1_ after using the intervention and not using the intervention, respectively (1 min 12 s ± 39 s, 28 s–5 min 45 s).

### 2.5 Analytical approach

#### 2.5.1 Feasibility

Activities with a completion rate below 20 % of the overall activity duration were excluded from the usage rate. Frequency statistics of usage and completion as well as of the acceptability questionnaire were calculated using IBM SPSS (Version 28.0.1.1) and Microsoft Excel (Microsoft Office Professional Plus 2016).

Interviews were transcribed by a professional transcription service (Amberscript Global B.V.) and checked for errors by a study team member. The interviews were analyzed using the structuring content analysis method by Mayring (Mayring, 2022) with MAXQDA (VERBI Software GmbH). Two study team members created a coding frame with seven deductive categories (13 subcategories), guided by the interview guide and material. First, one interview was coded by a study team member and the coding frame was adjusted where necessary, without adding inductive categories, and the remaining interviews were coded using the revised coding frame (see OSF Suppl. 6). A second team member then reviewed all of the codes and coded data, identified any discrepancies from the coding frame, and discussed them with the coder until consensus was reached.

#### 2.5.2 Momentary and daily measures

To test the immediate effect of EPIGRAM on momentary stress (H_1_) and fatigue (H_3_) during the self-reported stressful events, we created factors named Time (T_0_, T_1_) and Condition (intervention use, no intervention use) using EMA data from Phase 2 (intervention). Hypotheses 1 and 3 were tested using linear mixed models with random intercepts for observations nested in participants with planned contrasts, restricted maximum likelihood estimation, and fully crossed factors of Time and Condition. In addition, we included the time interval between T_0_ and T_1_ as within-person-centered (level-1) covariate to account for time differences (Enders and Tofighi, 2007). This deviates from our preregistered approach; however, adding this covariate is essential to account for the differing T_0_–T_1_ intervals across conditions. We then computed one-tailed planned contrasts at the T_0_ and T_1_ assessments. We adopted the Kenward-Roger approximation (Kenward and Roger, 1997) to estimate effective degrees of freedom in small samples for linear mixed models in order to obtain *F* tests for fixed effects. This was a deviation from our preregistration, in which we stated our intention of using Satterthwaite approximation, which provides χ^2^ values in Stata. We repeated the same approach for positive and negative affect as exploratory outcomes during the self-reported stressful events with two-tailed planned contrasts. We initially preregistered our intention to omit event-related entries when participants were instructed to use EPIGRAM but did not provide a follow-up measure. Despite two such occurrences, we decided to retain these cases, adhering to intention-to-treat principles (Gupta, 2011).

To assess effects on daily stress (H_2_) and fatigue levels (H_4_) from before to after the intervention, we computed linear mixed models with EMA data from Phase 1 (baseline) and Phase 3 (post), using the average scores from all assessments over the five consecutive days, and a dummy-coded variable and factor named Phase (baseline, post). The models again used restricted maximum likelihood estimation and the Kenward-Roger approximation to estimate effective degrees of freedom. We repeated the same approach for daily self-compassion and daily mindfulness as exploratory outcomes to investigate changes from before to after the intervention phase.

We analyzed the data using linear mixed models and plotted the results using R (Version 4.3.0) and Stata SE (Version 15).

## 3 Results

### 3.1 Participants

All participants were German-speaking and the majority were female (*n* = 20, 74 %). The mean age was 21.6 ± 1.9 years (range: 19–27) and all participants were studying psychology as their main degree. None of the participants were in full-time employment, while 18 (67 %) worked part-time. The sample was largely healthy, with three participants (11 %) reporting a chronic somatic disorder (e.g., asthma, neurodermitis), five (19 %) reporting a mental disorder (e.g., depression, anxiety disorder, eating disorder), and six (22 %) identifying as smokers. Participants reported elevated somatic symptom scores (PHQ-15: 8.2 ± 4.7, range: 0–19; compared to the norm values by Kocalevent et al., 2013) and mild depression scores (PHQ-9: 6.0 ± 3.8, range: 0–17; defined by Löwe et al., 2002) at baseline. Moreover, the sample reported elevated baseline levels of ongoing stress (PSS: 16.7 ± 6.8, range: 4–31; compared to the German norm values, Klein et al., 2016) and elevated baseline levels of fatigue (MFI general fatigue: 11.9 ± 3.9, range: 5–19; physical fatigue: 10.7 ± 4.5, range: 4–17; reduced activity: 11.2 ± 3.9, range: 5–19; reduced motivation: 8.6 ± 3.5, range: 4–19; mental fatigue: 11.0 ± 3.5, range: 4–18; compared to the German norm values, Schwarz et al., 2003). Participants' prior experiences with individual components of EPIGRAM were as follows: Seventeen (63 %) had experience with yoga, 13 (48 %) with relaxation activities, 11 (41 %) with meditation, six (22 %) with self-compassion activities, four (15 %) with time management training, one (4 %) with problem-solving training and one (4 %) with stress management programs/strategies. Six participants (22 %) had no prior experiences with any component of EPIGRAM.

### 3.2 Feasibility

#### 3.2.1 Usage and compliance

The overall completion rate for fixed assessments across the EMA period was 64 % (a total of 3151 data entries out of the scheduled 4860). Completion rates steadily decreased from Phase 1 to Phase 3 (Phase 1: 80 %, Phase 2: 65 %, Phase 3: 40 %). Regarding the event-related assessments, participants logged 174 stressful events (Phase 1: 41, Phase 2: 130, Phase 3: 3). On average, each participant logged 6.4 ± 4.3 (range: 0–18) stressful events throughout the study period. Examples for stressful events include “too much to do for my studies”, “ruminating thoughts when lying in bed”, “driving lesson”, or “family conflict”. In contrast, in the evening assessments, participants reported a total of 343 days with at least one stressful event (average per participant: 12.7 ± 7.5, range: 2–33). Based on the information reported in the evening assessment, on 64 % of the days, none of the stressful events that had occurred were logged, and on 12 % of the days, only some were logged, with percentages of unlogged events increasing from Phase 1 to Phase 3. In Phase 2, out of the 130 logged stressful events (T_0_: 62 intervention use, 64 no intervention use, 4 unknown), participants responded to 108 prompts at T_1_ (16 % missing). At T_1_, 54 data entries were available for both intervention use and no intervention use. OSF Suppl. 7 and 8 provide details on completion rates (including response durations and latencies) and characteristics of stressful events.

The intervention usage rates are shown in Table 2. On average, participants engaged in 24.1 ± 17.9 activities (range: 3–68), and 15.4 ± 15.1 (range: 0–55) activities when only considering the module activities (without the diary) for the self-initiated interventions. Three participants did not engage in any activities other than completing the diary. During (self-initiated) stressful event entries, participants engaged in an average of 2.2 ± 1.7 (range: 0–6) activities, with three participants not engaging in any activities.

**Table 2:** Usage rates for modules and activities.

Modules	Activity	Self-initiated intervention usage		Event-related intervention usage	
		*n* (*n* participants)	%^a^	*n* (*n* participants)	%^a^
1) Short-term stress management strategies	Psychoeducational introduction	23 (13)	6	/	
	Breathing meditation	3 (3)	1	8 (8)	14
	Progressive muscle relaxation	4 (4)	1	3 (3)	5
	Total number of activities	30	7	11	19
2) Meditation and relaxation	Psychoeducational introduction	29 (17)	7	/	
	Body scan	11^b^ (9)	3	4 (3)	7
	Breathing meditation	11^b^ (10)	3	2 (1)	3
	Focus meditation (sounds & thoughts)	17^b^ (9)	4	0	0
	Loving kindness meditation	8^b^ (7)	2	1 (1)	2
	Progressive muscle relaxation	4^b^ (4)	1	3 (3)	5
	Total number of activities	80	19	10	17
3) Music and nature sounds	Psychoeducational introduction	17 (8)	4	/	
	Personal study playlist	57 (13)	14	18 (12)	31
	Nature sound — gentle forest rain	8^b^ (4)	2	0	0
	Nature sound — thunderstorm at dusk	5 (4)	1	1 (1)	2
	Nature sound — summer day in the forest with stream	5^b^ (4)	1	3 (2)	5
	Nature sound — night ambience with crickets chirping	0	0	0	0
	Nature sound — beach day with seagulls	4^b^ (4)	1	1^a^ (1)	2
	Total number of activities	96	23	23	39
4) Yoga	Psychoeducational introduction	24 (13)	6	/	
	Yoga session 1	12 (10)	3		
	Yoga session 2	10 (7)	2		
	Yoga session 3	4 (3)	1		
	Yoga session 4	4 (3)	1		
	Yoga session 5	3 (2)	1		
	Total number of activities	57	14		
5) Problem solving	Psychoeducational introduction	27 (12)	6	/	
	Problem-solving training	9 (9)	2	/	
	Imagery: successful problem mastery	4 (4)	1	9 (9)	15
	Total number of activities	40	10	9	15
6) Time management	Psychoeducational introduction	19 (11)	5	/	
	Future vision	5 (5)	1		
	Prioritizing	7 (6)	2		
	Planning positive activities	5 (5)	1		
	Total number of activities	36	9		
7) Social support	Psychoeducational introduction	20 (10)	5	/	
	My social network	4 (4)	1		
	Practical tips for maintaining social contacts	9 (7)	2		
	Total number of activities	33	8		
8) Self-compassion	Psychoeducational introduction	22 (13)	5	/	
	How do I treat a valued person?	8 (7)	2	/	
	Hand on heart	6 (5)	1	2 (2)	3
	Letter of self-compassion^c^	4 (4)	1	/	
	Appreciation of the self	4 (4)	1	4 (4)	7
	Total number of activities	44	11	6	10
Extra: daily diary	Psychoeducational introduction	27 (18)	/	/	
	Review stressful and positive experiences of the day	208 (25)			
	Total number of activities	235			
All activities		651	/	59	100
Activities modules (without diary)		416	100		

*Note*. The information buttons to receive information about stress and stress management skills were pressed 42 and 18 times, respectively.

a Percentage relates to overall module activities, excluding the daily diary.

b This frequency number includes incomplete exercises (20–80 % of overall time). Overall, there were *n* = 13 incomplete exercises for the modules and *n* = 1 incomplete exercise for the event-related interventions.

c Option to read the letter was used twice.

#### 3.2.2 Usability and participant satisfaction

All of the participants completed the EMA period (no dropouts) and participated in the group interviews. However, one participant did not complete the online post questionnaire.

##### 3.2.2.1 Acceptability questionnaire

The majority of participants reported that, in their view, various components of the app could potentially reduce stress, especially relaxation activities (92 %), music listening (92 %), meditation (89 %), and yoga (89 %). Most participants found the EPIGRAM activities easy to use (69 %) and the app to be a suitable format (73 %). Activities were mostly experienced as helpful (58 %), pleasant (77 %), and enjoyable (77 %), and the majority of participants (73 %) would recommend components of EPIGRAM to a friend. However, 27 % of the participants often found engagement with the activities to be disruptive and 46 % reported feeling uncomfortable at times when engaging with the intervention in public (e.g., on public transport) or in front of others. Furthermore, over half of the participants (54 %) found the data entries to be disruptive and 62 % found the number of alarms to be too high (see Supplementary Tables S1 and S2 for further details).

##### 3.2.2.2 Group interviews

A total of 584 text segments were coded. In general, participants reported both long-term benefits (e.g., through engagement with their own stress) as well as short-term stress reduction (e.g., feeling calmer and more relaxed). However, some also reported a lack of long-term effects (e.g., due to insufficient time to practice) and short-term effects, which was largely related to difficulties in implementing the intervention during moments of acute stress, especially when faced with time-related stressors. The intervention was mainly rated as easy to use and to integrate into everyday life, although this depended on the context and activity. For example, activities such as listening to music and to nature sounds were described as easy to integrate, even while on public transport or during moments of acute stress, whereas yoga was seen as less flexible in terms of integration. The study app was described as user-friendly with a simple design. However, participants also mentioned technical problems with the app (e.g., lack of a back button within the app) as well as issues with the study smartphones and the app's visual appeal (see Supplementary Table S3 for further details).

### 3.3 Momentary and daily effects

#### 3.3.1 Immediate effects of EPIGRAM on stress and fatigue

**Fig. 2:**
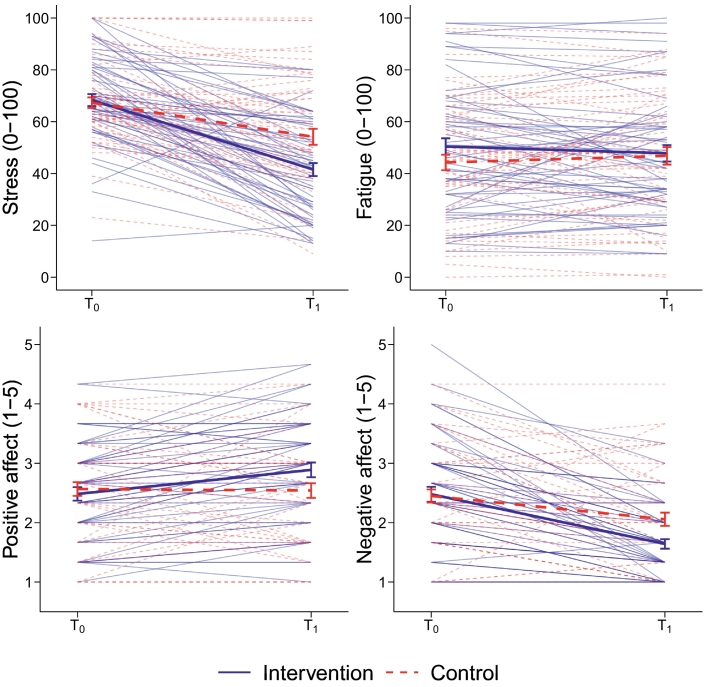
*Event-related changes for event-related stress, fatigue, and positive and negative affect.* Event-related trajectories by intervention and control condition. Thick lines indicate averages within each condition (±standard errors).

According to Hypotheses 1 and 3, we expected an immediate effect of EPIGRAM on momentary stress and fatigue during the self-reported stressful events. Before the intra-individual randomization to the event-related intervention use or no intervention use at T_0_, participants showed no significant differences in stress, *t*(198) = 0.26, *p* = .397, and fatigue, *t*(198) = −0.01, *p* = .498 (see Table 3 for means and standard deviations). Following the intra-individual randomization, participants showed a greater stress reduction from T_0_ to T_1_ when instructed to use EPIGRAM compared to no intervention use, as shown by the significant Time × Condition interaction for event-related stress, *F*(1, 188.14) = 10.14, *p* = .002 (see Table 3 and Fig. 2). Planned one-tailed contrasts at T_1_ revealed significantly lower stress when EPIGRAM was used compared to when no intervention was used, *t*(198) = 4.19, *p* < .001. However, using EPIGRAM did not lead to a greater decrease in fatigue levels from T_0_ to T_1_, *F*(1, 188.05) = 0.53, *p* = .466, compared to no intervention use.

**Table 3:** Means (SD) and immediate effects for event-related stress, fatigue, and positive and negative affect.

		Intervention use *M* (*SD*)		No intervention use *M* (*SD*)		Time		Condition		T_0_–T_1_ interval		Time × Condition	
		T_0_	T_1_	T_0_	T_1_	*F* (*df*, *ddf*)	*p*	*F* (*df*, *ddf*)	*p*	*F* (*df*, *ddf*)	*p*	*F* (*df*, *ddf*)	*p*
Secondary outcomes	Stress	68.3 (18.4)	41.5 (18.5)	67.3 (16.2)	54.2 (22.7)	95.82 (1, 188.14)	<0.001	7.98 (1, 202.97)	0.005	0.77 (1, 193.86)	0.381	10.14 (1, 188.14)	0.002
	Fatigue	50.4 (24.8)	47.8 (23.4)	44.0 (23.6)	46.8 (24.9)	0.00 (1, 188.05)	0.971	0.32 (1, 202.61)	0.574	2.39 (1, 193.64)	0.124	0.53 (1, 188.05)	0.466
Exploratory outcomes	Positive affect	2.5 (0.9)	2.9 (0.9)	2.6 (0.9)	2.5 (0.9)	4.15 (1, 186.82)	0.043	0.04 (1, 200.52)	0.844	4.41 (1, 191.97)	0.037	3.72 (1, 186.82)	0.055
	Negative affect	2.5 (1.0)	1.6 (0.6)	2.5 (0.9)	2.1 (0.8)	38.55 (1, 187.47)	<0.001	0.63 (1, 203.03)	0.427	0.93 (1, 193.44)	0.337	4.38 (1, 187.47)	0.038

*Note*. “Intervention use” and “no intervention use” refer to the conditions of the intra-individual randomization following each self-reported stressful event during Phase 2 (Intervention), *n* = 25 participants with *k* = 126 self-reported stressful events (62 intervention use, 64 no intervention use), excluding the 4 cases where the randomization is unknown. T_0_ refers to the item answered immediately after the stressful event, T_1_ to the item answered 20 min after the initial entry (no intervention use) or 20 min after the intervention was completed (intervention use). The intraclass correlation coefficients (*ICC*) for event-related stress, fatigue, and positive and negative affect were *ICC* = 0.167, *ICC* = 0.340, *ICC* = 0.342, and *ICC* = 0.220, respectively.

#### 3.3.2 Intermediate effects of EPIGRAM on stress and fatigue

According to Hypotheses 2 and 4, we expected a decrease in average daily stress and average daily fatigue from Phase 1 (baseline) to Phase 3 (post). While average daily stress decreased significantly from Phase 1 to Phase 3, *F*(1, 565.46) = 7.88, *p* = .005 (see Table 4 for means, standard deviations, and results), there was no significant change in daily fatigue, *F*(1, 568.94) = 0.17, *p* = .678.

**Table 4:** Means (SD) and intermediate effects for average daily stress and fatigue, mindfulness, and self-compassion.

		Phase 1 (baseline)	Phase 3 (post)	*F* (*df*, *ddf*)	*p*
		*M* (*SD*)	*M* (*SD*)		
Secondary outcomes	Stress	32.2 (22.8)	28.9 (19.6)	7.88 (1, 565.46)	0.005
	Fatigue	46.4 (25.7)	47.4 (25.0)	0.17 (1, 568.94)	0.678
Exploratory outcomes	Self-compassion	3.4 (0.7)	3.5 (0.6)	4.52 (1, 118.81)	0.036
	Mindfulness	2.5 (0.7)	2.7 (0.6)	5.15 (1, 122.89)	0.025

*Note*. Daily stress, fatigue, mindfulness, and self-compassion are obtained from averaging EMA entries during Phase 1 and Phase 3. The intraclass correlation coefficients (*ICC*) for daily stress, fatigue, self-compassion, and mindfulness were *ICC* = 0.215, *ICC* = 0.165, *ICC* = 0.291, and *ICC* = 0.111, respectively.

#### 3.3.3 Exploratory analyses

During the self-reported stressful events, at T_0_, participants reported no significant differences in positive affect, *t*(196) = 1.03, *p* = .151, or negative affect, *t*(198) = −0.67, *p* = .252 (see Table 3 for means and standard deviations). After using EPIGRAM, the results indicated no statistically significant increase in positive affect, *F*(1, 186.82) = 3.72, *p* = .055, but significantly lower negative affect, *F*(1, 187.47) = 4.38, *p* = .038, compared to no intervention use (see Table 3 and Fig. 2). Planned two-tailed contrasts indicated that differences between using EPIGRAM and no intervention use at the post-test were significant for negative affect, *t*(198) = 1.92, *p* = .028.

Finally, daily self-compassion and mindfulness showed significant increases from the baseline to the post phase, as indicated by significant effects of Phase for self-compassion, *F*(209.3) = 4.52, *p* = .036, and mindfulness, *F*(1,122.89) = 5.15, *p* = .025 (see Table 4 for means, standard deviations, and results).

## 4 Discussion

The main goal of this mixed methods pilot study was to explore the feasibility of an individually customizable ecological momentary stress management intervention. We additionally aimed to gain initial insights into the immediate effect of the intervention following stressful events, as well as the pre- to post-intervention effect in everyday life. Our results indicate the overall feasibility of the intervention and reduced feasibility of the study design. They further demonstrate that the intervention was successful in reducing both event-related and daily stress (supporting Hypotheses 1 and 2), but not fatigue (in contrast to Hypotheses 3 and 4). The intervention also led to improvements in event-related affect and daily self-compassion and mindfulness from baseline (Phase 1) to post (Phase 3).

### 4.1 Feasibility

The general compliance with the assessment schedule in the present study (64 %) was slightly lower compared to other ecological momentary assessment studies (average of 72 % in 32 studies reviewed, De Vries et al., 2021), which might indicate that the assessment schedule was too burdensome and led to decreased motivation, as reported by some participants in the acceptability questionnaire. The perceived assessment burden may also have influenced overall participant satisfaction. Future studies should therefore reduce the assessment burden and could consider strategies to increase participants' adherence to the fixed assessment schedule (e.g., assessment intervals with adaptable reminders at times that align with participants' daily routines).

The number of logged stressful events (6.4 per person) was similar to comparable studies (e.g., 7.7 for chronically stressed individuals, Feneberg and Nater, 2022). However, participants experienced more stressful events than they had actually logged during the day, as reported retrospectively in the evening assessments (average of 12.7 days with at least one stressful event). While some discrepancy between these reports is expected, given that stressful events in daily life do not always allow immediate logging (e.g., the smartphone is unavailable or the situation requires a specific action), the discrepancy observed here is higher than in other studies (Feneberg and Nater, 2022). However, the main reasons for not logging stressful events (time-related and circumstantial barriers) are similar to those reported in previous research (Feneberg and Nater, 2022). One possible explanation for this higher discrepancy might lie in the above-mentioned decreasing compliance with the assessment schedule. In addition, participants logged fewer stressful events per day during the intervention phase compared to the baseline phase. This may reflect a decrease in motivation or an anticipated burden associated with conducting an intervention, as logging a stressful event could prompt an intervention and thus discourage reporting. Future studies could address this by allowing participants to actively dismiss or snooze the start of the intervention in response to stressful events. This may also provide valuable insight into the optimal time point to intervene in response to daily stressful events, which is still an open question in the field (Smyth and Ebner-Priemer, 2025). Furthermore, future studies could explore alternative approaches to identifying stressful events in daily life, such as using physiological stress markers (e.g., wearable-reported heart rate variability, Garland et al., 2023), which may also reduce participant burden.

Intervention usage rates (89 % for event-related and self-initiated intervention) were similar to comparable studies (Feneberg and Nater, 2022; D'Adamo et al., 2023; Nguyen-Feng et al., 2019). The most frequently used activities were passive, familiar, and brief activities (e.g., music listening, nature sounds, meditation, relaxation techniques), especially when the current circumstances were not ideal for using the intervention (e.g., on public transport, in the presence of others). These activities appear to be most suitable for self-administered EMIs and event-related intervention components and thus warrant further exploration. Although music was the preferred activity in our study and has shown good feasibility in other EMIs (Feneberg and Nater, 2022), our exploratory analysis did not demonstrate it to be more effective in reducing event-related stress than other activities (see OSF Suppl. 11). To confirm this, further investigation with a larger sample for each EPIGRAM activity and sufficient power is necessary.

In addition, future studies should explore which stressful events are more or less suitable for momentary digital stress management, and which situational circumstances support or hinder its use in daily life. As our results and other studies (Feneberg and Nater, 2022) show, some stressful events (e.g., time-related stressors) and situational circumstances (e.g., the presence of others) pose challenges for momentary digital stress management and may require the implementation of stress management at an earlier or later time point, or using different strategies or tools.

### 4.2 Momentary and daily effects

#### 4.2.1 Immediate effects

Stress and negative affect decreased when using the event-related intervention components, which is in line with previous studies investigating the impact of event-related interventions on different health outcomes (Smyth and Heron, 2016; Wang and Miller, 2020). The effects are also supported by our qualitative data, with participants reporting that they felt more relaxed, calmer, or less stressed after using the event-related intervention. However, these findings are biased by the low rates of logging stressful events in relation to the number of stressful events actually experienced, as discussed above, and should therefore be interpreted with caution. In addition, sensitivity analyses indicate the influence of the day of the week (weekday vs. weekend) on the immediate effects of fatigue and affect (for details see OSF Suppl. 13). Future studies are needed to confirm and further investigate these effects as well as explore best-practice implementations of event-related intervention components.

#### 4.2.2 Intermediate effects

The intervention was effective in reducing daily stress, in line with findings from previous studies investigating stress management interventions and EMIs targeting stress (Schulte-Frankenfeld and Trautwein, 2022; Amanvermez et al., 2020; Heber et al., 2017). Interestingly, the pre- to post-intervention effects on daily stress were not replicated with regard to ongoing stress (PSS scores, for details see OSF Suppl. 12), potentially indicating that the assessment period or intervention duration was too short to yield significant changes in ongoing stress (according to previous research, interventions with a duration of 5–8 weeks revealed the strongest effects (Amanvermez et al., 2020; Heber et al., 2017). Furthermore, this discrepancy may reflect recall biases influencing retrospective questionnaire scores, thus underlining the importance of EMA (Trull and Ebner-Priemer, 2013). Future EMIs will be necessary to explore the event-related and daily effects in a large-scale randomized controlled trial.

Our findings revealed significant pre- to post-intervention changes in daily levels of mindfulness, which corresponds to previous EMA studies on digital mindfulness-based interventions (Mirabito and Verhaeghen, 2023). The pre-to-post change in the present study was very small and should therefore not be overinterpreted. However, this finding is reflected in our qualitative data, which showed that some participants reported an increase in some facets of mindfulness (Baer et al., 2006) in the group interviews, for example by reporting more awareness regarding their own stressors and stress experiences or a mental and emotional distancing from stressors. Since mindfulness has been shown to moderate the link between daily hassles and affect (Junça-Silva et al., 2023), future studies should explore the potential mediating and moderating effects of daily mindfulness on stress in EMIs. Analogously, self-compassion, which is connected to mindfulness (Biehler and Naragon-Gainey, 2022; Dreisoerner et al., 2021a), changed slightly but significantly from pre- to post-intervention and could be explored as a possible mediator or moderator of daily stress in EMIs in future studies.

The present study did not reveal a decrease in fatigue, either in terms of daily levels from pre-to post-intervention or regarding event-related fatigue. This finding does not appear to be influenced by floor effects, as VAS scores ranged around 50 and the MFI baseline scores were above the norm values of the German general population (Schwarz et al., 2003). For daily levels of fatigue, a longer intervention or later post-assessment might have been necessary for observable change, as other studies reported significant reductions in fatigue, for example in cognitive-behavioral (Malouff et al., 2008) or mindfulness-based interventions (Chayadi et al., 2022). However, as the majority of studies investigating fatigue have focused on clinical samples, questions concerning effective fatigue management strategies in the general population remain open. It is also possible that our intervention was not suitable for reducing fatigue. It may be necessary to specifically target fatigue in the intervention, for example by specifically addressing it in psychoeducational elements. Similar to the present results, this could increase awareness of fatigue and, as a result, may also lead to reductions in fatigue. Further exploration is needed to understand how stress management interventions might improve fatigue in everyday life.

### 4.3 Strengths and limitations

This study is the first to investigate an individually customizable ecological momentary stress management intervention that combines a traditional digital health application with an event-related component. The mixed-methods design, combining EMA with standardized questionnaires, acceptability questions, and group interviews, facilitated an in-depth examination of feasibility while investigating the effects of the intervention on stress and fatigue from multiple perspectives.

Furthermore, the investigated student sample showed elevated baseline levels of ongoing stress (compared to the German PSS norm values, Klein et al., 2016) and fatigue (compared to the German MFI norm values, Schwarz et al., 2003). Using the PSS-10 cut-off score of ≥13 to indicate chronic stress, as employed in previous literature (Feneberg and Nater, 2022), 70.4 % of our sample would be considered as chronically stressed. The high psychological burden of the investigated sample highlights the need for feasible and effective stress management interventions for university students.

However, some limitations of the present study must be acknowledged. Major limitations lie in the small sample size and the lack of a control group, which restrict the reliability of our quantitative results. In addition, decreasing compliance with EMA may have reduced data quality by increasing missingness and potential measurement bias. Regarding the effects of the event-related intervention, we attempted to enhance the reliability of our findings by using an intra-individually randomized design. Nevertheless, all quantitative results should only be interpreted as initial trends regarding the effect of the intervention, which need to be confirmed in a large-scale randomized controlled trial. For the interpretation of the main research question of feasibility, however, the sample size is sufficient (Teresi et al., 2022). The variability in the T_0_–T_1_ interval may further restrict the reliability of immediate intervention effects. While we included the T_0_–T_1_ interval as a covariate to address this, future studies should attempt to keep the interval consistent between conditions.

Additionally, the homogeneity of the investigated student sample reduces the generalizability of the findings, as all participants were recruited from a single university course. Furthermore, the additional face-to-face support (introductory session, question and answer group sessions) may have influenced the results, potentially limiting their generalizability to a purely digital setting. In addition, the involvement of study team members in teaching the university course, along with the lack of anonymity in the group interviews, may have influenced participants' study behavior and responses (e.g., trend towards positive feedback).

### 4.4 Outlook

High levels of ongoing stress pose a risk for poor mental health and the development of various somatic and mental health disorders (Schneiderman et al., 2005). Therefore, it is crucial to develop and investigate flexible digital stress management applications that can be feasibly implemented in everyday life and foster coping with both acute stressful events and ongoing stressors. By providing flexible, low-threshold coping strategies in daily life, EMIs and event-related intervention components may become extremely useful in mental health care (Piot et al., 2022), for example as initial steps in stepped-care approaches or as add-on treatments to standard care. Our pilot study provided strong evidence for the feasibility of our ecological momentary stress management intervention EPIGRAM and showed its potential to reduce event-related and daily stress in everyday life. Building on these promising results, future studies should explore the effect of our intervention in large-scale randomized controlled trials, using an adapted EMA assessment schedule in a more heterogeneous sample, and additionally consider the effects of the intervention on biological stress markers (e.g., cortisol). It would further be especially interesting to investigate other chronically stressed groups or (sub-)clinical populations with stress-related conditions. In view of their considerable potential, future research should focus on innovations and best practices for a feasible and effective implementation of event-related intervention components as mental health interventions.

## Author contributions

HT: conceptualization, methodology, study conduction, analysis, writing original draft, visualization, project administration. AD: methodology, study conduction, analysis, writing original draft, visualization, reviewing and editing manuscript. KS: conceptualization, methodology, reviewing and editing manuscript. RM: conceptualization, methodology, reviewing and editing manuscript. UN: conceptualization, methodology, reviewing and editing manuscript, supervision.

## Declaration of Generative AI and AI-assisted technologies in the writing process

During the preparation of this work the authors used ChatGPT (version GPT-4, OpenAI) in order to improve the readability and language of the manuscript. After using this tool, the authors reviewed and edited the content as needed and take full responsibility for the content of the published article.

## Funding

This research did not receive any specific grant from funding agencies in the public, commercial, or not-for-profit sectors.

## Declaration of competing interest

The authors declare no competing interests.

## Data Availability

The de-identified data and code supporting the findings of this study are openly available on OSF (https://osf.io/zfh8q/files) under the folder “3_Code and dataset”. Study materials are also accessible on OSF (https://osf.io/zfh8q/files) under the folder “2_Materials and supplements”. The entire OSF project can be accessed at doi:10.17605/OSF.IO/ZFH8Q.

## Appendix A Supplementary data

Supplementary tables Image 1

## Footnotes

Appendix A Supplementary data to this article can be found online at https://doi.org/10.1016/j.invent.2025.100901.
