# Supplementary material for: Feasibility and preliminary effects of an individually customizable ecological momentary stress management intervention: A mixed methods pilot study
Source: Internet Interv. 2026 Jan 13;43:100901. doi: 10.1016/j.invent.2025.100901 (PMC12857363; doi:10.1016/j.invent.2025.100901)
Supplement: Supplementary file 1 — Supplementary tables [file mmc1.docx]

**Table S1**

*Acceptability Ratings on a Dichotomous Scale*

| **ID** | **Item** | **Agree** |
| --- | --- | --- |
| 1 | I am satisfied with the study as a whole. | 19 (73%) |
| 2 | The app was difficult to use. | 2 (8%) |
| 3 | The data entries in the app were generally disturbing. | 14 (54%) |
| 4 | I often found the self-assessments via app to be disturbing. | 11 (42%) |
| 5 | I often found the engagement with the intervention activities (modules) to be disturbing. | 7 (27%) |
| 6 | The number of alarms per day was burdensome. | 16 (62%) |
| 7 | The intervals between the different alarms were too short. | 9 (35%) |
| 8 | I would have answered questions about my stress experience via the app more than three times a day. | 7 (27%) |
| 9 | There were differences in my daily routine and state of mind between workdays and weekends. | 18 (69%) |
| 10 | I could express my experienced feelings and changes with the given terms in the app.^a^ | 12 (46%) |
| 11 | The questions for the data entries in the app were easy to understand. | 25 (96%) |
| 12 | The duration of the data entries in the app was reasonable. | 23 (88%) |
| 13 | I sometimes felt uncomfortable entering data in the app in public (e.g., riding the bus, lecture, work) or in front of others.^a^ | 9 (35%) |
| 14 | I sometimes felt uncomfortable engaging in intervention activities (modules) in public (e.g., riding the bus, lecture, work) or in front of others.^a^ | 12 (46%) |
| 15 | I found it difficult to make data entries in the app during moments of increased stress (stress events). | 25 (96%) |
| 16 | There were situations when I was stressed (stress events) which I did not report.^a^ | 25 (96%) |
| 17 | I changed my behaviour as a result of my study participation.^a^ | 16 (62%) |
| 18 | My study participation led to increased introspection/self-observation. | 21 (81%) |
| 19 | The 45 days of study participation were representative of my everyday life.^a^ | 18 (69%) |
| 20 | I omitted entries in the app, e.g., because I forgot them.^a^ | 23 (88%) |
| 21 | I answered questions dishonestly (e.g., because they made me feel uncomfortable). | 0 (0%) |
| 22 | There were single, personally important events that I did not want to log in the app.^a^ | 11 (42%) |
| 23 | I would have needed more technical support in order to use the app. | 0 (0%) |
| 24 | I would have needed more support from the study team in order to engage with the intervention activities. | 0 (0%) |
| 25 | I had technical problems with the app that I have not yet discussed with the study team.^a^ | 1 (4%) |

**Note.** ^a^Additional information was provided in an open answer format. Those data were analysed as part of the descriptive summary of additional feedback (handwritten notes, question and answer sessions). *n* = 26.

**Table S2**

*Acceptability Ratings on a Likert Scale*

| **ID** | **Item** | **(Strongly) Disagree** | **Partly** | **(Strongly) Agree** | ***M*** | ***SD*** | **Range** |
| --- | --- | --- | --- | --- | --- | --- | --- |
| 1 | The intervention activities were easy to conduct. | 2(8%) | 6 (23%) | 18 (69%) | 4.0 | 1.0 | 2-5 |
| 2 | I felt confident in conducting the activities. | 2 (8%) | 5 (19%) | 19 (73%) | 4.0 | 0.9 | 2-5 |
| 3 | The self-administered conduction of the activities via the app was a suitable format for me. | 4 (15%) | 3 (12%) | 19 (73%) | 4.0 | 1.2 | 1-5 |
| 4 | I think most people would quickly learn the activities and be able to use them in everyday life. | 4 (15%) | 7 (27%) | 15 (58%) | 3.8 | 1.2 | 1-5 |
| 5 | I generally found the activities to be helpful. | 1 (4%) | 10 (39%) | 15 (58%) | 3.8 | 1.0 | 1-5 |
| 6 | I generally found the activities pleasant. | 2 (8%) | 4 (15%) | 20 (77%) | 3.9 | 1.0 | 1-5 |
| 7 | I generally enjoyed the activities. | 1 (4%) | 5 (19%) | 20 (77%) | 4.1 | 0.8 | 2-5 |
| 8 | I feel like I have learned something by doing the activities. | 3 (12%) | 7 (27%) | 16 (62%) | 3.9 | 1.2 | 1-5 |
| 9 | I would recommend the activities to a friend. | 2(8%) | 5 (19%) | 19 (73%) | 4.2 | 1.1 | 1-5 |
| 10 | I plan to continue conducting activities similar to those presented in the study. | 3 (12%) | 5 (19%) | 18 (69%) | 3.9 | 1.1 | 1-5 |
| 11 | Do you think relaxation activities can reduce individual stress levels (independently of the activities presented in the app)? | 0 (0%) | 2 (8%) | 24 (92%) | 4.7 | 0.6 | 3-5 |
| 12 | Do you think meditation can reduce stress individual stress levels (independently of the activities presented in the app)? | 0 (0%) | 3 (12%) | 23 (89%) | 4.7 | 0.7 | 3-5 |
| 13 | Do you think listening to music can reduce individual stress levels (independently of the activities presented in the app)? | 1 (4%) | 1 (4%) | 24 (92%) | 4.6 | 0.8 | 2-5 |
| 14 | Do you think yoga can reduce individual stress levels (independently of the activities presented in the app)? | 2(8%) | 1 (4%) | 23 (89%) | 4.4 | 1.0 | 1-5 |
| 15 | Do you think self-compassion can reduce individual stress levels (independently of the activities presented in the app)? | 0 (0%) | 7 (27%) | 19 (73%) | 4.0 | 0.8 | 3-5 |
| 16 | Do you think time management can reduce individual stress levels (independently of the activities presented in the app)? | 3 (12%) | 3 (12%) | 20 (77%) | 4.2 | 1.2 | 1-5 |
| 17 | Do you think problem-solving training can reduce individual stress levels (independently of the activities presented in the app)? | 2(8%) | 7 (27%) | 17 (65%) | 4.0 | 1.2 | 1-5 |
| 18 | Do you think social support can reduce individual stress levels (independently of the activities presented in the app)? | 0 (0%) | 6 (23%) | 20 (77%) | 4.4 | 0.9 | 3-5 |

**Note.** Possible answers range from 1 "do not agree at all" to 5 "fully agree". *n* = 26.

**Table S3**

*Results from the Online Group Interview*

| **Category** | | **Frequency of coded segments** | **Summary of main content** | **Exemplary quote** |
| --- | --- | --- | --- | --- |
| **ID** | **Description** | **n (%)** |  |  |
| **Effects of the intervention** | | | | |
| U1-1 | Improvement of long-term stress management competency | 50 (9) | increased awareness of and engagement with stress, acquiring a wider range of stress management techniques implementable in the long-term. | “But, uh, maybe there are some long-term effects, possibly, because this app has made me more generally aware or at least a bit more conscious of my own stress perception. And even though I no longer actively have these modules directly available, uh, I notice that it has somewhat changed my general, my overall perspective on my own experience of stress over time, and some of that definitely remains.” *(group 4, 30.01.23)* |
| U1-2 | No improvement of long-term stress management competency | 15 (3) | lack of long-term effects, mainly attributed to insufficient practice time, lack of routine in implementing app activities, and a difficulty retaining app learnings | “Um, so I found that this long-term, um, didn't really show up as long-term positive consequences. Um, because I no longer had access to the modules, […] I couldn't use it anymore and couldn't integrate it into my daily life. And, um, exactly for this reason, it unfortunately didn't help much. Now, at the moment, um, I don't feel the stress reduction that I felt during the usage.” *(group 3, 23.01.23)* |
| U2-1 | Short-term stress reduction | 57 (10) | positive experiences such as feeling more relaxed, calm, and gaining distance or new perspectives on stress-inducing problems | “But it was like during the breathing exercise I calmed down and relaxed” *(group 1, 09.01.23)* |
| U2-2 | No short-term stress reduction | 31 (5) | the intervention was unfeasible, felt ineffective, or even increased momentary stress levels during moments of increased stress, especially time-related stress | “But in acute stressful situations, I noticed that I couldn't focus on the exercise because I was just caught up in my problem, and I am mostly stressed in situations due to time pressure.” *(group 1, 09.01.23)* |
| **Ease of use and integration into everyday life** | | | | |
| U3-1 | Ease of use of the intervention | 47 (8) | wide range of diverse activities, the flexibility of the intervention, the simplicity of conducting activities (especially music and nature sounds), the low-threshold of the app | “So, um, I think that was kind of the idea of the app, that it (inaudible) that there was a colorful bouquet of modules, some meant for short-term issues and others for long-term stress reduction. And, um, I think it's great that the app offered such a wide range of options, both for short-term and long-term.” *(group 4, 30.01.23)* |
| U3-2 | Difficulties with the intervention | 79 (14) | certain activities being too time-consuming (e.g., yoga), writing exercises demanding high effort, lack of personal appeal in specific activities | “Yes, I also remember that it was sometimes very hindering for me when I knew I had to write something, for example with the problem-solving strategies or the daily review, I think it was called, that I knew, okay, now I have to write this down on paper again.” *(group 1, 09.01.23)* |
| U4-1 | Integration into daily life | 52 (9) | for certain activities while being in public or in transit and during moments of increased stress (i.e., music and meditation activities), for certain activities or strategies (e.g., self-reflection) in their daily routines | “So, I found, um, actually music and nature sounds, um, the easiest to integrate and the most intuitive to use, and therefore also the most helpful.” *(group 2, 16.01.23)* |
| U4-2 | Difficulties with integration into everyday life | 59 (10) | some activities were impractical to complete while being in public or in transit (e.g., yoga) or when interruptions were possible and likely,  difficulties integrating the intervention into their daily routines (e.g., due to lack of time) and integrating it in moments of increased stress | “And then I always thought, okay, if I do an exercise now, it will basically take even more time away from me.” *(group 1, 09.01.23)* |
| **Usability of the app** | | | | |
| U5-1 | Ease of use of the study app | 9 (2) | user-friendliness and simple design | “So, I didn't find it complicated at all. It was really very easy to use.” *(group 1, 09.01.23)* |
| U5-2 | Difficulties with the study app | 44 (8) | technical problems (e.g., lack of back-button within the app), issues with the study smartphones and the app design (e.g., lack of visual appeal) | “[…] for me, a back button was definitely missing. Sometimes I clicked by mistake, and then I was in some exercise and had to click through it like that. […] But as a user, it was just a bit cumbersome.” *(group 2, 16.01.23)* |
| **Satisfaction with the study design** | | | | |
| U6-1 | Positive experiences with the study design | 17 (3) | enhanced self-reflection through daily measurements and the psychoeducation provided in the seminar | "Um, and I actually found this whole knowledge about self-compassion and what components it consists of, um, I found that very helpful. Developing such a modality, just thinking like that. I found that much more helpful, um, yes, um, to reduce my stress." *(group 4, 30.01.23)* |
| U6-2 | Negative experiences with the study design | 36 (6) | event-based measurements were experienced as hindering in stressful situations, disruptive alarms, fixed measurement time points did not align with participants’ routines | “[…] what bothered me a bit was that the measurements, um, they bothered me a bit that it was standardized time." *(group 3, 23.01.23)* |
| **Additional category** | | | | |
| U7 | Suggestions & Ideas | 88 (15) | further app and intervention development (e.g., more short exercises, individually selectable duration of music listening) | "Um, and I somehow wished that there would be options that are shorter. So not like they have to be at least ten minutes long." *(group 4, 30.01.23)* |

*Note.* Exemplary quotes have been translated from German.
